# The Behavioral Intervention with Technology for E-Weight Loss Study (BITES): Incorporating Energy Balance Models and the Bite Counter into an Online Behavioral Weight Loss Program

**DOI:** 10.1007/s41347-020-00181-4

**Published:** 2020-11-27

**Authors:** Carly M. Goldstein, Stephanie P. Goldstein, Diana M. Thomas, Adam Hoover, Dale S. Bond, J. Graham Thomas

**Affiliations:** 1 The Weight Control and Diabetes Research Center, The Miriam Hospital, Providence, RI 02903, USA; 2 Department of Psychiatry and Human Behavior, Alpert Medical School, Brown University, Providence, RI 02903, USA; 3 Department of Mathematical Sciences, The United States Military Academy, West Point, NY 10996, USA; 4 Holcombe Department of Electrical and Computer Engineering, Clemson University, Clemson, SC 29634, USA

**Keywords:** Weight loss, Technology, Obesity management, Feeding behavior, Eating

## Abstract

This study evaluated feasibility and acceptability of adding energy balance modeling displayed on weight graphs combined with a wrist-worn bite counting sensor against a traditional online behavioral weight loss program. Adults with a BMI of 27–45 kg/m^2^ (83.3% women) were randomized to receive a 12-week online behavioral weight loss program with 12 weeks of continued contact (*n* = 9; base program), the base program plus a graph of their actual and predicted weight change based on individualized physiological parameters (*n* = 7), or the base program, graph, and a Bite Counter device for monitoring and limiting eating (*n* = 8). Participants attended weekly clinic weigh-ins plus baseline, midway (12 weeks), and study culmination (24 weeks) assessments of feasibility, acceptability, weight, and behavioral outcomes. In terms of feasibility, participants completed online lessons (*M* = 7.04 of 12 possible lessons, *SD* = 4.02) and attended weigh-ins (*M* = 16.81 visits, *SD* = 7.24). Six-month retention appears highest among nomogram participants, and weigh-in attendance and lesson completion appear highest in Bite Counter participants. Acceptability was sufficient across groups. Bite Counter use (days with ≥ 2 eating episodes) was moderate (47.8%) and comparable to other studies. Participants lost 4.6% ± 4.5 of their initial body weight at 12 weeks and 4.5% ± 5.8 at 24 weeks. All conditions increased their total physical activity minutes and use of weight control strategies (behavioral outcomes). Although all groups lost weight and the study procedures were feasible, acceptability can be improved with advances in the technology. Participants were satisfied with the online program and nomograms, and future research on engagement, adherence, and integration with other owned devices is needed. ClinicalTrials.gov Identifier: NCT02857595

## Introduction

Online behavioral weight loss programs, while not always as efficacious as gold standard in-person treatment, often produce clinically relevant weight losses and are increasingly utilized as a scalable means of intervention (Neve et al. 2010). However, participants completing these programs may be susceptible to suboptimal weight loss if they fail to adhere to recommendations for a reduced-calorie diet, physical activity, and self-monitoring. Innovative methodologies that provide more immediate and personalized feedback on behavior change efforts can potentially improve the efficacy of online behavioral weight loss programs while maintaining scalability.

Data visualization is one such tool that provides an opportunity for patients to obtain nearly immediate feedback on weight and weight-related behaviors (Martin et al. 2015). Many commercially available technology-based tools, like smartphone applications, facilitate self-monitoring of weight-related behaviors, which is a cornerstone of online behavioral weight loss programs (Butryn et al. 2011). These tools typically use visualization to immediately display an individual’s information back to them and can include progress towards a weight goal, daily caloric and macronutrient intake, and physical activity minutes (and provide an opportunity for individuals to have this information at their fingertips).

According to self-regulation theory, individuals may benefit most from self-monitoring when it is paired with data visualization tools that directly compare their progress to an expected outcome in a meaningful, understandable way (Rapp and Cena 2014). One strategy to harness data visualization as a more powerful behavior change tool is to involve nomograms (i.e., graphs) that depict expected weight change produced via caloric restriction (Martin et al. 2016; Martin et al. 2015). These nomograms are shaped by a validated dynamic mathematical model that predicts weight change based on an individual’s personalized parameters including age, sex, and daily calorie goals (Martin et al. 2015; Thomas 2013). The graph depicts a “zone” of expected body weight change that is based on adherence to a calorie goal (Martin et al. 2015). Weekly body weight measurements are plotted on the nomogram and compared to the predicted weight loss zone; the objective is to use this information to guide participants to make necessary dietary changes to return their weight within the nomogram’s bounds. If a participant’s weight loss falls outside the zone, one can be confident that they are not achieving optimal adherence to the recommendations for caloric restriction and/or physical activity, and therefore further behavior change will be necessary. This is an important innovation because many weight-related behaviors, and caloric intake in particular, are difficult to self-monitor accurately (Dhurandhar et al. 2015). Intake is often grossly underestimated (Johnson et al. 2005), which can lead to confusion and frustration when dietary goals appear to be met but weight loss does not result.

Preliminary research demonstrates that nomograms effectively indicate potential weight loss failure. Being out of the predicted weight loss zone is a sensitive predictor of not achieving a 5% weight loss 1 year post-baseline (Thomas et al. 2015a). Furthermore, previous research that added the nomogram to a low-calorie diet produced greater weight loss than a health education program alone over 12 weeks (Martin et al. 2015). Nomograms could therefore serve as a useful adjunct to the recommendations for self-weighing in online behavioral weight loss, but the feasibility of this approach has never been evaluated. One limitation of the nomogram is that it only *identifies* suboptimal adherence but does not provide tools or resources to *improve* it.

Wearable technologies that automatically track eating behavior and provide support for limiting intake are a logical supplement to weight loss nomograms. The Bite Counter is a wrist-worn device that uses an inertial measurement unit to infer number of bites taken based on the wrist-roll motion that occurs as food is brought to the mouth (Scisco et al. 2011). Bites are a useful metric as they correlate with caloric intake (Salley et al. 2016), and bite counting has been used as an intervention tool in prior work. The DIET Mobile study is the only other behavioral weight loss trial using the Bite Counter that is at least 6 months duration, which is representative of standard behavioral weight loss programs. That study randomized half the participants to use a smartphone app to manually track dietary intake and half to receive the Bite Counter; both received twice-weekly weight loss podcasts. Participants tracked daily bites and attempted to stay within personalized prescribed daily bite goals that were adjusted based on weekly weight losses (Turner-McGrievy et al. 2017b). This study found that both the Bite Counter and manual tracking via app produced clinically significant weight loss when combined with educational podcasts, though participants that received the Bite Counter lost less weight than the smartphone app group but achieved greater physical activity (Turner-McGrievy et al. 2017b). The DIET Mobile study provides preliminary evidence that the Bite Counter, combined with personalized bite goals, may support clinically meaningful weight loss across the typical length of an effective behavioral weight loss program.

Given the prior research on both nomograms and the Bite Counter, we hypothesized that these tools could be utilized together to enhance adherence to online behavioral weight loss program recommendations. Nomograms can assist individuals with interpreting their personal weight trajectories, with clear demarcation of when they have deviated from expected program goals. In conjunction, the Bite Counter can be programmed with personalized bite goals for participants when the nomogram indicates they may be at risk for weight loss failure. Therefore, the combination of the nomogram and Bite Counter has potential to provide timely, personalized feedback. However, it is unknown if combining these strategies is feasible and acceptable to individuals participating in online weight loss programs. It is unclear whether individuals will engage with these tools sufficiently enough to impact weight loss or whether the added burden may diminish adherence and outcomes.

The purpose of the current pilot study is to describe the use of weight loss nomograms with and without the Bite Counter and bite goals in a group of adults receiving a validated online behavioral weight loss program. We sought to evaluate the feasibility and acceptability of the Bite Counter and the nomogram combined in the context of a standard online behavioral weight loss program. The online weight loss program alone was included as a comparison condition. Feasibility and acceptability (primary outcome) of the online weight loss program, nomogram, and Bite Counter were assessed via compliance with treatment procedures and satisfaction with treatment and technology, respectively. Weight-related behaviors, such as weight control strategies and physical activity, as well as weight were also assessed as secondary outcomes. Given that the primary objective of this pilot study was to establish feasibility and acceptability across all three study conditions, no a priori hypotheses were specified. We present descriptive information of our primary and secondary outcomes across all three study conditions.

## Methods

### Subjects

Participants were English-speaking adults aged 18–70 years with a body mass index (BMI) of 27–45 kg/m^2^. Participants were required to have access to the Internet via a computer and self-reported basic computer skills. They were required to report willingness to use a computer and other technology-based tools (that would be provided to them at no cost) to assist them with weight loss. The exclusion criteria were medical contraindication for weight loss or unsupervised exercise; self-reported heart condition or chest pain during periods of activity or rest; loss of consciousness per the Physical Activity Readiness Questionnaire (Thomas et al. 1992); currently pregnant or breastfeeding, or intention to become pregnant in the next 6 months; planning to relocate out of the geographical area in the next 6 months; cognitive or physical limitations that prevent use of a computer or Bite Counter; participating in a study at the research center within the last 2 years; currently participating in a commercial weight loss program or participating in a commercial weight loss program in the prior 6 months; ≥ 5% weight loss in the prior 6 months; history of a clinically diagnosed eating disorder excluding binge eating disorder; history of a surgical procedure to cause weight loss; currently taking weight loss medication; has received medical treatment for cancer in the previous 6 months; or likely inability to adhere to the study procedures or unlikely to benefit from weight loss due to a health condition or life circumstance as determined by the principal investigator (e.g., current substance dependence or terminal illness).

### Procedure

The recruitment target for this pilot trial was 30 individuals allocated at a 1:1:1 ratio to each intervention group. The three intervention groups were (a) online behavioral weight loss program and in-person weekly weigh-ins; (b) online behavioral weight loss program with brief in-person weekly weigh-ins including charting weight loss on a nomogram; and (c) online behavioral weight loss program with briefly weekly in-person weigh-ins including charting weight loss on a nomogram and provision of a Bite Counter with an alarm set to a bite count limit based on the nomogram. Weekly weigh-ins were led by an assigned masters- or doctoral-level interventionist and kept to < 10 min. The interventionist did not provide comprehensive weight loss counseling. All conditions were 24 weeks total.

The senior author developed the 1:1:1 randomization scheme such that participants were randomized in permuted blocks of nine. The randomization was stratified for an equal balance of genders across groups. The primary outcomes were retention, mean number of online lessons completed, weigh-in appointment attendance, and self-reported satisfaction ratings of the intervention. Bite Counter use (only among participants who received the Bite Counter), weight loss, physical activity, and weight control strategies were secondary outcomes.

Participants were recruited from local advertisements and a research center waiting list. Interested individuals completed a phone screen to determine eligibility, and eligible individuals attended a group orientation session in which the study procedures were explained in detail, their eligibility was confirmed, and they were given the opportunity to consent to participate. Individuals who consented then completed baseline questionnaires, their body weight and height were measured in a private room, and they were provided with the physical activity monitor (described below), which they wore on their upper arm for 1 week during waking hours.

Participants returned to the research center for their randomization visit after wearing the armband for 7 days. During this kickoff session, they learned their randomization assignment; were given goals for weight loss, diet, and physical activity (described below); and were taught basic strategies for getting started in the online weight loss program (e.g., how to self-monitor, navigating the online platform). They then received a 12-week online weight loss program followed by 12 weeks of continued contact (described below). Participants completed follow-up assessments at 12 and 24 weeks. All three assessments were identical except participants reported on their use and satisfaction of their assigned interventions at 12 and 24 weeks. The number of lessons completed was extracted from the online platform at the final assessment.

Participants were compensated $25 at 12 and 24 weeks for completing the assessment procedures. All informed consent procedures and study protocol were approved by the Institutional Review Board of The Miriam Hospital and conducted at The Miriam Hospital in Providence, RI, USA.

### Interventions

Program goals were introduced in the kickoff session, which was an hour-long session designed to teach the fundamental skills of behavioral weight loss. They were prescribed an overall weight loss goal of 10% of their baseline body weight (Butryn et al. 2011) with a target of losing 1–2 pounds (0.45—0.91 kg) per week. Once a participant reached their 10% weight loss goal, they were supported in weight maintenance or, if they wanted to continue to lose weight and their BMI was > 27, they pursued additional weight loss. They were instructed to eat a balanced diet of 1200 kcal and < 40 g of dietary fat (i.e., 30% calories from fat) if their baseline body weight was < 250 pounds (113.40 kg) or 1500 kcal and < 50 g of dietary fat if their baseline body weight was ≥ 250 pounds. They were encouraged to engage in 50 min weekly of moderate-to-vigorous physical activity in bouts of at least 10 min beginning in week 1, gradually increasing to 200 min weekly by weeks 11–12. They were taught behavioral strategies for increasing their exercise and lifestyle physical activity and to record their daily kilocalorie and fat intake, body weight, and physical activity. They were informed that while day-to-day fluctuations in body weight are normal, their weight should generally trend downward. Though their weight loss may have slowed in weeks 13–24, participants were asked to focus on weight loss throughout all 24 weeks unless they hit their 10% weight loss goal making their BMI < 27.

All participants received the Rx Weight Loss Program, a previously validated online behavioral 12-week weight loss program conducted over the Internet (Thomas et al. 2015b). Based on strategies taught in the Diabetes Prevention Program and Look AHEAD trials, participants were given 12 weekly 15-min interactive lessons focused on behavior change to support weight management (Diabetes Prevention Program Research, G 2002; Look AHEAD Research Group 2006) through the secure platform. Lesson topics included stimulus control, problem solving, self-monitoring, healthy diet, exercise, weight regain prevention, and strategies for weight loss maintenance. The platform also provided forms for participants to record and submit daily calories, fat grams, physical activity minutes, and body weight, due every Sunday evening. A new lesson and algorithm-generated feedback on self-monitoring records were released each Monday for the first 12 weeks. Participants retained access to the Rx Weight Loss Program through their participation in the entire program (24 weeks). In weeks 13–24, participants met with their interventionist for continued contact. At each brief weigh-in, they were offered support and targeted problem solving.

Participants assigned to the nomogram group received everything in the base program detailed above. During each weigh-in appointment, they also received a weight loss nomogram. The “zone” that they were trying to keep their body weight within is created by fitting an upper and a lower curve through the mean absolute error from validated differential equations (Thomas et al. 2011). The interventionists explained the zone of projected weight loss and the nomogram during the kickoff session and as needed during individual sessions. During the weigh-ins, body weight was plotted on the nomogram, and participants were asked to compare their measured weight to the zone of expected weight loss given adherence to their goal for caloric intake. If their weight fell above the zone of expected weight loss, potential contributors to nonadherence were briefly discussed, including lapses from the dietary plan, possible sources of unintentional underreporting of intake, and unintentional over-estimation of physical activity. This was framed as a supportive discussion in which difficulties with self-monitoring or adhering to dietary and physical activity recommendations are normal and understandable.

Participants randomized to the Bite Counter group received all of the intervention components that the nomogram group received. In addition, they were provided with a 2nd-Generation Bite Counter smartwatch (Bite Technologies, Pendleton, SC) to be worn on their dominant wrist, charging cord with wall adapter, and a user manual created for the study. All participants received devices that displayed time, number of bites taken that day, and step count at their kickoff visit. They were taught how to use the Bite Counter during this appointment, and the interventionist confirmed their understanding with practice and observation plus clarification when needed. The Bite Counter can function as both a monitor and an intervention tool, and it is activated by a button press at the start and end of an eating episode (Dong et al. 2012). Participants were instructed to wear the Bite Counter during all waking hours and to press the on/off button at the beginning of every meal, snack, or consumption of a beverage with calories (e.g., water was excluded when consumed alone). After completing their eating episode, they were instructed to press the on/off button again to complete the bite recording process. Bite counts could be stored on the device for up to approximately 4 weeks of regular use. When participants came to their weekly weigh-in appointments at the research center, the data were uploaded, their clinic weight was plotted on the nomogram in collaboration with the interventionist, and they compared their actual weight to the zone of expected weight loss. If a participant presented to the clinic with 2 consecutive weeks with weights above the zone of expected weight loss, an upper daily bite goal was calculated to produce weight change in the middle of the zone of projected weight loss. Upper daily bite goals were determined by correlating average daily bites in all weeks before they exceeded the projected weight loss zone, current average daily bites, and weight change. At the next scheduled weigh-in appointment, after downloading the last week’s Bite Counter data, the interventionist programmed the Bite Counter with a daily bite goal alarm. The alarm beeped when they reached their goal, indicating that they should stop eating at that time. Participants were allowed to request for the alarm to sound up to five bites before their limit as a warning if they preferred. Bite goals typically remained the same throughout the program once assigned (visible as a watch view), and alarms were not discontinued once they were initiated. Interventionists supported participants in stopping eating closer to the alarm when participant data regularly demonstrated continued eating past the alarm and that week’s weight loss was suboptimal.

### Measures

All participants provided demographic information and medical history by questionnaire. Weight was measured in kilograms to the nearest 0.1 kg using a medical-grade, calibrated, digital body weight scale. At each assessment (baseline, 12 weeks, and 24 weeks), participants provided two weight measurements in light clothing without shoes. Standing height was measured in millimeters with a stadiometer. BMI was calculated at baseline to verify study eligibility.

Feasibility and acceptability were assessed in two domains: compliance with treatment procedures and satisfaction with treatment and technology. The online weight loss program recorded the number of online lessons each user completed. Interventionists recorded the number of scheduled weekly weigh-ins (out of 24) that each participant attended. Additionally, at the study completion (24 weeks), participants reported their satisfaction with the program components they received by completing a feedback questionnaire designed specifically for this study using a 7-point Likert scale with higher ratings indicating greater satisfaction. The proportion of days the device was used to record at least two eating episodes was calculated for participants who received a Bite Counter. The Bite Counter also collected the average number of bites per day and number of logged eating episodes, and the research team recorded the week in which Bite Counter users received a bite goal.

Weight loss and behavioral outcomes in this study included changes in weight, physical activity, and utilization of weight control strategies (which also includes questions regarding dietary change). Weight change was reported in kilograms and calculated as percent change in baseline body weight after 12 and 24 weeks. Total minutes of weekly moderate-to-vigorous physical activity were measured by the SenseWear armband (Body Media, Pittsburgh, PA) at all three assessments. By wearing the armband on the upper right triceps, the device’s sensors provide an accurate estimate of energy expenditure attributed to physical activity (Jakicic et al. 2004). At all three assessments, participants completed the validated Weight Control Strategies Scale (Pinto et al. 2013). Participants report how often they engage in specific dietary choices, self-monitoring strategies, physical activity, and psychological coping in the last month through 30 items.

### Statistical Analysis

Data were analyzed with IBM SPSS 25 (IBM Corp, Armonk, NY). Descriptive information was used to accomplish the primary aim of this study (establish feasibility and acceptability of utilizing the nomogram and Bite Counter with the online behavioral weight loss intervention). Descriptive statistics included means and standard deviations of continuous variables and frequencies and percentages for categorical variables. Missing weight, physical activity, and weight control strategy use data were imputed using the conservative baseline observation carried forward method. Given the small sample size, and the goals of this pilot study, no a priori hypotheses were specified and therefore no inferential analyses were conducted. This is consistent with best practices for reporting pilot studies’ results (Thabane et al. 2010).

## Results/Findings

### Demographic Information

**Fig. 1:**
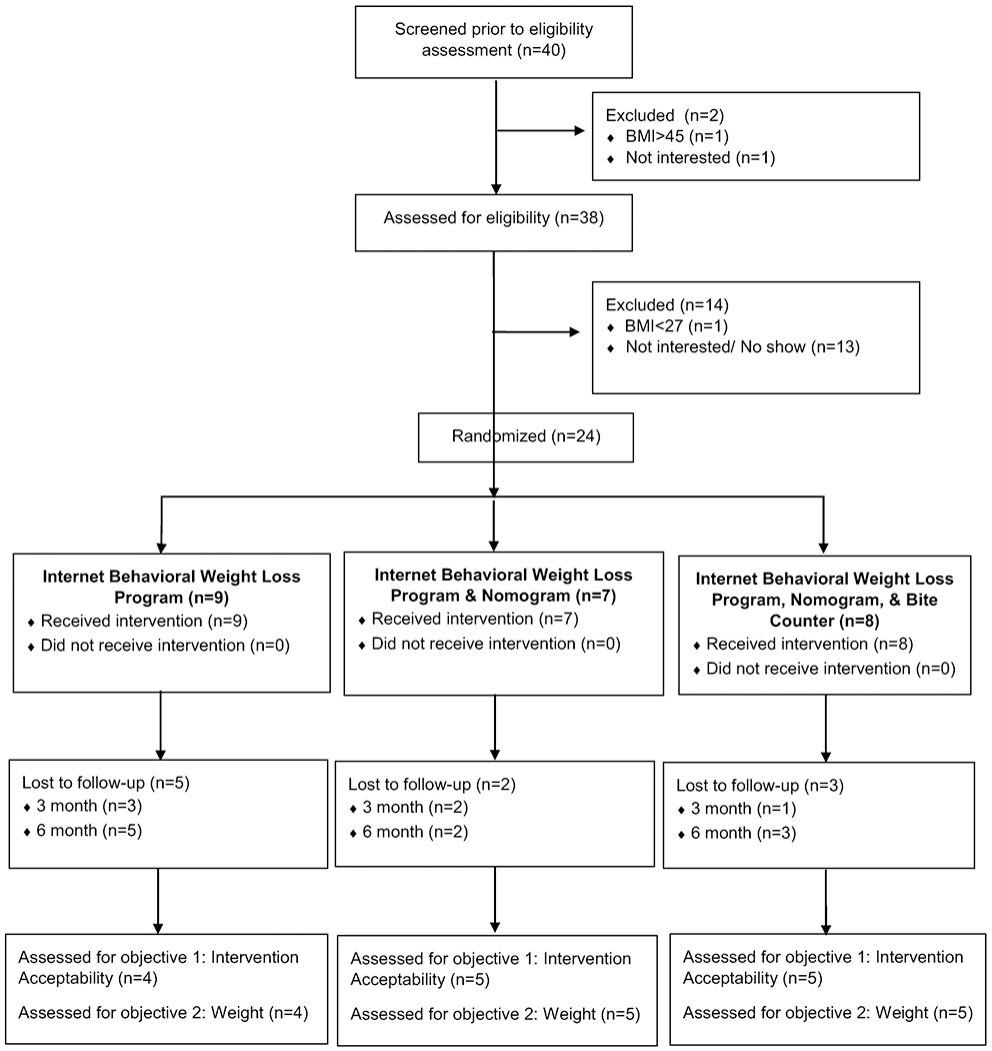
BITES CONSORT participant flow

Participants were randomized to the online behavioral weight loss condition (*n* = 9), online behavioral weight loss plus nomogram condition (*n* = 7), and online behavioral weight loss plus nomogram and Bite Counter condition (*n* = 8). A CONSORT diagram that depicts participant flow can be found in Fig. 1. Participants were mostly female, middle-aged, White, and non-Hispanic. Table 1 displays participant characteristics from the overall sample and by treatment condition.

### Feasibility and Acceptability

#### Compliance with Treatment Procedures

**Fig. 2:**
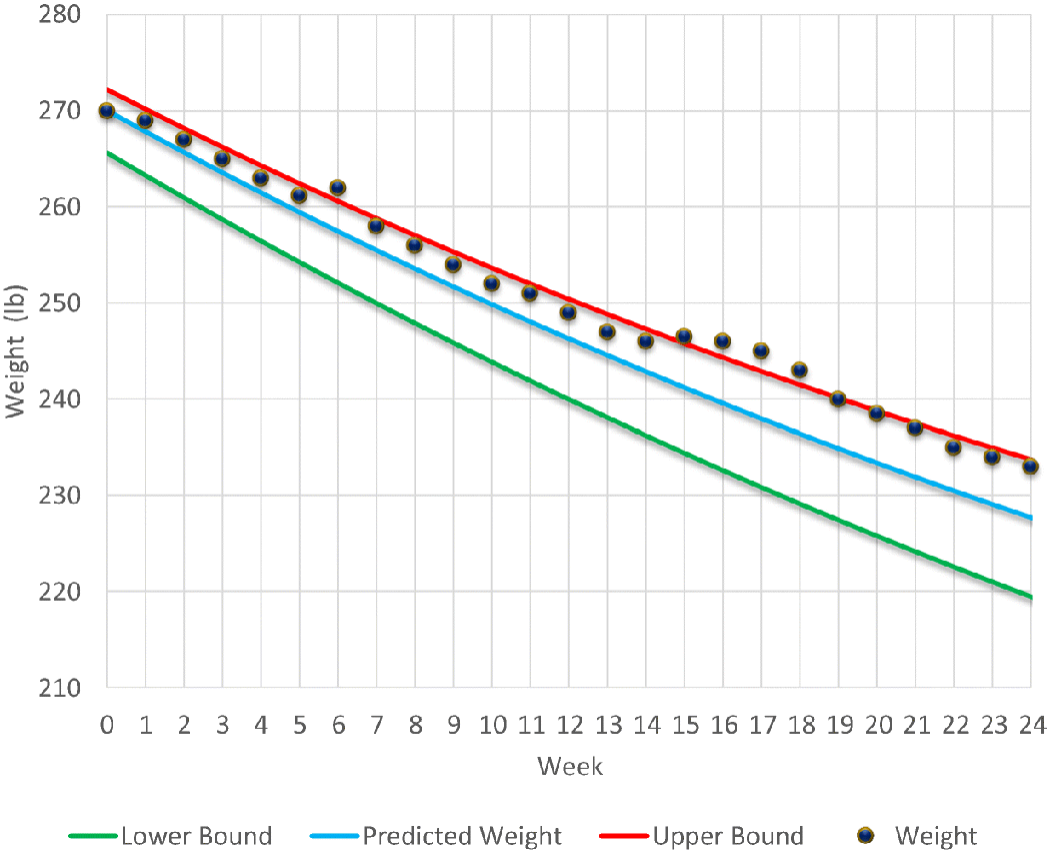
A sample weight loss nomogram depicting the zone of adherence and weight loss plotted on the graph from baseline to week 24. *Note*. This is the nomogram for a 65” tall 60-year-old female. The starting weight is 270.00 lbs. and BMI is 44.9. The bite goal is provided in week 17 after suboptimal weight loss in weeks 15 and 16. Optimal weight in week 15 is 234.35–245.77 and in week 16 is 232.56–244.30. Week 15 weight is 246.50 and week 16 weight is 246.00. Although the weight in week 6 is above the upper limit, no bite goal is provided because there has not been two consecutive weeks of suboptimal weight loss. The 10% weight loss goal (270.00 lbs) should occur at week 17, but in actuality was achieved at week 18. However, the participant would be encouraged to continue losing weight as her BMI would be > 27

Per Table 1, study retention across treatment appeared to be higher in the groups receiving the nomogram and the nomogram with Bite Counter. Of the 12 online behavioral weight loss sessions, participants completed an average of 7.04 lessons (*SD* = 4.02). Participants in the online behavioral weight loss condition completed an average of 6.22 (*SD* = 4.21) lessons, the group that also received the nomogram completed an average of 6.14 lessons (*SD* = 4.34), and the group that received all intervention components completed an average of 8.75 lessons (*SD* = 3.41). Participants attended, on average, 16.81 (*SD* = 7.24) of the 24 scheduled weigh-ins (17.28 ± 6.84 weigh-ins attended in the group that only received the online program v. 14.14 ± 9.04 weigh-ins attended in the group that also received the nomogram v. 18.75 ± 5.94 weigh-ins attended in the group that received the online behavioral weight loss program, nomogram, and Bite Counter). For participants using the Bite Counter, a day of wear-time was considered valid if at least two eating episodes were recorded during the day, which is considered best practice for measuring adherence based on a previous investigation (Turner-McGrievy et al. 2019). There were 653 days of valid Bite Counter wear-time across 8 participants that received the Bite Counter, which corresponds to an average of 47.8% (*SD* = 31.63%) of study days with valid Bite Counter wear-time. Across days with valid wear-time, participants averaged 119.75 bites (*SD* = 56.34) and 3.25 eating episodes (*SD* = 1.00) per day. Out of the eight participants randomized to receive a Bite Counter, six received bite goals after falling above the nomogram’s zone of expected weight loss. One remained in the zone of expected weight loss and never triggered a bite goal, and one discontinued their participation after 2 weeks of being out of the zone for reasons unrelated to receiving a bite goal. On average, participants who received a bite goal received it after 14 weeks of participation after losing 6.21% of their baseline body weight. On average, participants experienced their first dyad of consecutive out-ofzone weeks during weeks 12 and 13 while still working towards their 10% weight loss goal. See Fig. 2 for an sample weight loss nomogram.

#### Satisfaction with Treatment and Technology

Participant satisfaction with the online behavioral weight loss program and the technology-based tools (i.e., nomogram. Bite Counter) was assessed via patient feedback questionnaire, with higher ratings (up to 7) indicating greater satisfaction. Overall, participants in all study conditions were very satisfied with the online behavioral weight loss program (*M* = 6.32, *SD* = 0.99); this level of satisfaction was similar across the group that online received the online program (*M* = 6.00, *SD* = 1.41), the group that also received a nomogram (*M* = 6.30, *SD* = 0.84), and the group that received the online program, nomogram, and Bite Counter (*M* = 6.60, *SD* = 0.89). Participants were highly satisfied with the nomogram in the group that received the nomogram with the online program (*M* = 6.41, *SD* = 0.69) and in the group that also received a Bite Counter (*M* = 5.53, *SD* = 1.12). Participants were least satisfied with the Bite Counter (*M* = 3.87, *SD* = 2.00). Common issues with the Bite Counter included difficulty wearing the device during work activities, redundancy when the participant was already used to wearing a wrist watch or other smartwatch, and forgetting to press the button at the beginning or end of an eating episode (see “Discussion”).

### Weight Loss and Behavioral Outcomes

Descriptive information on weight loss, physical activity, and weight control strategy use can be found in Table 2 and represent intent-to-treat sample (baseline observation carried forward). On average, participants lost 4.59% of their starting body weight (*SD* = 4.46) at 12 weeks and ultimately lost 4.53% (*SD* = 5.79) at 24 weeks. Of note, participants that received the nomogram with or without the Bite Counter appeared to continue to lose or maintain weight in weeks 13–24, whereas the group that only received the online program did not. Participants in all treatment conditions increased their total physical activity minutes throughout the study, although it was notable that the group that received the Bite Counter appeared to be less active at baseline than the two groups that did not, and this trend continued throughout the program. Participants in all treatment conditions reported increasing their use of weight control strategies in the first 12 weeks, and these gains appeared to be maintained at 24 weeks.

## Discussion

This is one of the first studies to report the feasibility and acceptability of using the Bite Counter device and weight loss nomograms in adult participants losing weight a 24-week online behavioral weight program. The potential of these methodologies is significant, as the field needs innovative ways to improve outcomes in online behavioral weight loss programs. Despite conventional concerns that these technologies can incur additional burden, adding the nomograms and Bite Counter was feasible and acceptable as measured by study retention, attendance at weigh-ins, and adherence to key aspects of treatment such as viewing online weight loss lessons in this small pilot trial. In fact, participants who also received the nomogram had 60.81% higher 6-month study retention (71.4% retained) than the online treatment only group (44.44% retained) and 14.24% higher 6-month retention than the Bite Counter group (62.50% retained). Participants who received the nomogram and Bite Counter had 40.77% higher 6-month retention than the online treatment only group. It is important to note that there is insufficient statistical power to justify inferential statistics to compare these differences, so larger sample sizes should be a priority in future studies. On average, participants completed approximately 60% of the online lessons, which is similar to previous studies (Unick et al. 2019). Previous studies using this online system have set a benchmark of at least 7 of 12 lessons completed to be considered good adherence (Wing et al. 2010), which was the average lesson completion rate in this study across groups. Notably, Bite Counter participants attended 32.60% more weigh-ins (18.75 of 24 weigh-ins attended on average) and completed 42.51% more lessons (8.75 of 12 lessons watched on average) than those who received the nomogram with the Rx Weight Loss Program (14.14 weigh-ins attended and 6.14 average lessons viewed). Bite Counter participants completed 8.51% more weigh-ins and 40.68% more lessons than individuals who only received the online weight loss program (Rx Weight Loss-only participants attended an average of 17.28 weigh-ins and watched 6.22 lessons). Future research will need to determine if this pattern is clinically and statistically significant.

The present study provides valuable insight into the process of including Bite Counters in behavioral weight loss programs, and it is only the second trial to use Bite Counters across 6 months. Participants who received the online program plus the nomograms and Bite Counter were only adherent to the device instructions (wearing and pressing the button during eating episodes) approximately 50% of the days, though this appears to be slightly higher than another 24-week study that demonstrated Bite Counter use on 40% of days (Turner-McGrievy et al. 2017b). Participants in the present study consumed almost 20 more bites per day than participants in another study using the Bite Counter (Turner-McGrievy et al. 2017a), which represents a difference of 17.17% and approximately 220 additional calories consumed per day in this study since most participants in this study were women (Scisco et al. 2014). The number of eating episodes was similar to a small study exploring the Bite Counter as a weight loss tool (Wilson 2014).

Overall, participants were generally satisfied with the online program and nomograms. Although participants who received the Bite Counter reported high overall program satisfaction (6.60/7), they expressed lower satisfaction with the Bite Counter (3.87/7), which may have contributed to suboptimal compliance with daily device use. Previous work has demonstrated that participant preference of an intervention is not necessarily related to better weight loss outcomes (Borradaile et al. 2012). Sometimes participants lose more weight with their less-preferred intervention (Burke et al. 2008). While future research should aim to improve intervention technology usability, participant satisfaction may not entirely predict weight loss or health outcomes. Anecdotally during the final assessment, one participant reported that her healthcare job did not allow her to wear the device while performing medical procedures on patients (partially due to concerns about repeatedly using medical-grade sanitizers on the device), though she still found the device useful. She reported wearing the device outside of patient contact and wearing the device while eating and drinking. Other participants who were skilled craftspeople also reported being unable to wear the device during some work tasks for fear of breaking the device. Some reported that the device felt burdensome if they already wore a watch or another digital health tracker. Since the Bite Counter needs to be worn on the dominant wrist used for eating (and for most people this is the right wrist) and includes a watch function, it can replace a wrist watch. However, wrist watches are typically worn on the nondominant wrist, putting this device on the “wrong” wrist for individuals who only want to wear a Bite Counter or a wrist watch. This goes against social norms and contributes to reduced acceptability. In response to feedback from this study and other Bite Counter projects, the Bite Counter device was transitioned to an algorithm that can now be downloaded on other digital smartwatches, many of which are more durable and easier to sanitize. However, some smartwatches also cost more. Nevertheless, this challenge to acceptability remains and should be further evaluated with a larger population of working adults who download the algorithm onto other commercially available smartwatches.

Another factor that may have contributed to lower satisfaction is that the Bite Counter requires a button press to initialize and finish recording bite activity, which may be a barrier to compliance. Some participants reported forgetting to press the button before or after (or both) during at least one eating episode within the program, though many reported that this happened regularly (e.g., monthly, weekly). Importantly, other projects are working to eliminate the need for the button press to enable continuous eating detection (Sharma et al. 2020). While more research will be needed, these innovations will likely improve acceptability. Overall, this proof-of-concept research illustrates the feasibility of incorporating the Bite Counter (with personalized bite goals and alarms) along with weight loss nomograms in online behavioral weight loss programs, and future research should explore the efficacy of using this evolving technology in larger trials that are fully powered for tests of efficacy. Additional trials using the Bite Counter as part of long-term weight loss programs are ongoing, which will add to the growing literature on wearable technology to support health behavior change. The investigation of wearable technologies in conjunction with robust evidence-based programs is important because these tools are not likely to be primary drivers of sustainable health behavior change on their own (Patel et al. 2015).

We measured weight loss and behavioral outcomes (physical activity and use of weight control strategies). Average weight loss (4.59% at 12 weeks and 4.53% at 24 weeks) appeared slightly lower than weight losses seen in previous studies using the Rx Weight Loss Program (5.8% at 12 weeks and 5.6% at 24 weeks; Ross et al. 2019; Thomas et al. 2015b). However, this prior research benefited from more staff contact (e.g., phone calls), participant support, and collaboration with the participants’ primary care providers. Participants who received the online program alone in this study lost weight in the first 12 weeks and maintained that weight loss in the next 12 weeks. However, individuals that received the nomogram with or without the Bite Counter continued to lose or virtually maintain weight across weeks 13–24. Future research with larger samples may evaluate whether the nomogram can help participants continue to lose weight after the novelty of the program wears off and they approach their first 5% loss (ideally after the first 12 weeks). This is especially valuable in longer programs, which tend to produce better weight loss and weight maintenance if participants remain engaged. The nomogram may be used as a “rescue” intervention when participants begin to consistently show suboptimal weight loss. Although the nomogram was feasible to use, future research must determine the best combination of tools (e.g., base program, Bite Counter, and nomogram combined) to achieve this effect. Future research should determine if the Bite Counter or nomogram can be used to prevent the typical pattern of weight regain after weight loss (Kramer et al. 1989).

Moreover, all participants reported increasing physical activity and weight control strategy use, further demonstrating the feasibility of this approach for supporting weight management. It should be noted that participants appeared to increase their weight control strategy use from baseline to 12 weeks, but they did not continue to implement additional strategies (beyond what was observed at the midpoint assessment) in the second half of the study. This is explained by the program structure, which involves more intensive treatment in the first 12 weeks with weekly lessons, self-monitoring, and feedback. Notably, physical activity minutes were generally high at baseline, thus leaving less room for improvement. It should be noted that the intervention was not designed to further increase physical activity in weeks 13–24 beyond the goal of 150 min per week set in the first half of the program. Nevertheless, a larger trial can confirm if these interventions increase physical activity and use of weight control strategies as evidenced here. A longer trial is also needed to determine if these improvements persist and are maintained during longerterm weight loss maintenance.

This study has several strengths including assessing participants’ satisfaction with novel behavioral weight loss program interventions (i.e., the Bite Counter and nomogram). In this study, participants reported lower satisfaction with the Bite Counter, although advancements in the technology described above will likely improve satisfaction. Participants were highly satisfied with the nomogram, which provides important insights into data visualization tools that participants find straightforward and easy to use. This is also one of the first studies to evaluate using the Bite Counter and nomograms over 24 weeks, which is more representative of typical behavioral weight loss programs (Wadden et al. 2020). This descriptive pilot study provides preliminary evidence of the feasibility and acceptability of these approaches as well as guidance for researchers and clinicians interested in supplementing behavioral weight loss programs with these or similar tools, continuing to improve the feasibility and acceptability of these tools, and improving health outcomes. Future studies may aim to improve the implementation of these novel tools to maximize their effectiveness and aim to reach a minimum threshold of weight loss before proceeding to a larger trial (Freedland et al. 2019). Future studies may also consider the best combinations of these tools with other interventions to produce maximal weight loss within acceptable limits of participant burden, such as in an optimization design (Collins et al. 2014).

This study also has important limitations. The sample was largely White, non-Hispanic, female, educated, affluent, and married. Though this demographic is prevalent in weight loss research and treatment, the results have limited generalizability to other populations also affected by obesity. Future research may target weigh-in appointment attendance, which was 70.00% in this study, through financial incentives or other methods. Of note, advancements in technology (e.g., Bite Counter apps, web-conferencing, Bluetooth scales) make it possible to conduct these procedures remotely and thus may improve adherence to study recommendations to record body weight daily via appointments or self-monitoring logs. The analysis of weight change followed the intent-to-treat principle in which missing data were imputed using the baseline observation carried forward method. Additionally, with a small sample size, this study is underpowered to detect between-groups differences. Consequently, the aims were focused on exploring feasibility and acceptability rather than establishing efficacy. Lastly, the Bite Counter should ideally be worn during all waking hours; however, some individuals may have jobs that precluded them from wearing such devices during at work. These individuals may have been less likely to enroll, less likely to remain in the program, or had poorer device-wearing adherence.

These results suggest that using weight loss nomograms and a wrist-worn eating tracker is feasible and acceptable for adults receiving online behavioral weight loss treatment, though satisfaction with the bite counting device could be improved. Requiring a button press to start and end eating detection and lack of integration with other prevalently used technologies (e.g., smartphones) are targets for future research; improvement in these areas will likely increase user satisfaction and adherence with using the Bite Counter. Participants were very satisfied with the nomogram, and participants who received the nomogram with and without the Bite Counter continued losing or maintaining weight in weeks 13–24 as opposed to the group who did not receive these tools. Provision of the nomogram and Bite Counter did not appear to reduce retention. With the aforementioned improvements to the user experience of the Bite Counter, adding the nomogram alone or in conjunction with the Bite Counter may help participants continue losing weight after the first 12 weeks of an online behavioral weight loss program. Future improvements to the user experience and capitalizing on technological and mathematical advances will be necessary to build the most effective suite of scalable intervention options that support healthy weight management across heterogeneous participant populations.

## Figures and Tables

**Table 1:** Participant characteristics

	Total (*n* = 24)		Online program alone (*n* = 9)		Online program with nomogram (*n* = 7)		Online program with nomogram and Bite Counter (*n* = 8)	
	Mean	SD	Mean	SD	Mean	SD	Mean	SD
Age	49.75	8.89	47.89	9.16	50.29	11.6	51.38	6.39
BMI (kg/m^2^)	35.17	5.21	34.17	4.19	36.08	6.37	35.61	5.66
Categorical participant characteristics								
	Total (*n* = 24)		Online program alone (*n* = 9)		Online program with nomogram (*n* = 7)		Online program with nomogram and Bite Counter (*n* = 8)	
	Number	%	Number	%	Number	%	Number	%
Sex								
Female	20	83.3	7	77.8	6	85.7	7	87.5
Male	4	16.7	2	22.2	1	14.3	1	12.5
Race								
White	20	83.3	8	88.9	6	85.7	6	75.0
Black or African American	2	8.3	0	0.0	1	14.3	1	12.5
Other	2	8.3	1	11.1	0	0.0	1	12.5
Ethnicity								
Not Hispanic or Latino	22	91.7	8	88.9	6	85.7	8	100.0
Hispanic or Latino	2	8.3	1	11.1	1	14.3	0	0.0
Education								
Junior high school or less	1	4.2	0	0.0	1	14.3	0	0.0
Attended or graduated from high school or earned GED	3	12.5	0	0.0	1	14.3	2	25.0
Vocational training (beyond high school)	3	12.5	2	22.2	0	0.00	1	12.5
Some college (< 4 years)	6	25.0	3	33.3	1	14.3	2	25.0
College or university degree	9	37.5	2	22.2	4	57.1	3	37.5
Graduate or professional education (e.g., MBA, MS, MA, PhD, MD, JD)	2	8.3	2	22.2	0	0.0	0	0.0
Marital status								
Married	17	70.8	5	55.6	5	71.4	7	87.5
Separated	1	4.2	0	0.0	0	0.0	1	12.5
Divorced	2	8.3	0	0.0	2	28.6	0	0.0
Widowed	1	4.2	1	11.1	0	0.0	0	0.0
Never married	1	4.2	1	11.1	0	0.0	0	0.0
Living with a partner (not married)	2	8.3	2	22.2	0	0.0	0	0.0
Income								
Under $50,000	1	4.2	0	0.0	1	14.3	0	0.0
$50,000–$99,999	13	54.2	4	44.4	4	57.1	5	62.5
$100,000 or higher	7	29.1	4	44.4	2	28.6	1	12.5
Prefer not to answer	3	12.5	1	11.1	0	0.0	2	25.0
Employment status								
Employed full-time	18	75.0	7	77.8	5	71.4	6	75.0
Employed part-time	3	12.5	1	11.1	1	14.3	1	12.5
Student status	3	12.5	1	11.1	1	14.3	1	12.5
Retention								
3 months	18	75.0	6	66.7	5	71.4	7	87.5
6 months	14	58.3	4	44.4	5	71.4	5	62.5

**Table 2:** Weight loss, physical activity, and weight control strategy use

	Total			Online program alone			Online program with nomogram			Online program with nomogram and bite counter		
	Baseline	3 mos	6 mos	Baseline	3 mos	6 mos	Baseline	3 mos	6 mos	Baseline	3 mos	6 mos
Weight loss (kg) *M*(*SD*)	--	4.59 (4.46)	4.53 (5.79)	--	3.99 (4.96)	2.96 (4.69)	--	4.61 (5.09)	5.91 (7.56)	--	5.26 (3.73)	5.10 (5.55)
Percent weight loss *M*(*SD*)	--	4.63 (4.27)	4.41 (5.36)	--	4.47 (5.17)	3.10 (4.94)	--	4.27 (4.47)	5.47 (6.62)	--	5.12 (3.43)	4.97 (5.03)
Total PA minutes/week *M*(*SD*)	111.46 (105.14)	125.21 (115.25)	135.67 (137.66)	138.89 (112.08)	173.33 (147.39)	186.67 (140.71)	105.00 (109.38)	110.71 (103.46)	120.00 (177.48)	86.25 (99.42)	83.75 (66.96)	92.00 (82.89)
WCSS scores *M*(*SD*)	1.42 (0.57)	2.45 (0.76)	1.99 (0.88)	1.50 (0.53)	2.42 (0.92)	1.94 (0.92)	1.39 (0.57)	2.41 (0.89)	2.19 (0.99)	1.35 (0.66)	2.52 (0.48)	1.88 (0.81)
